# E2814 Mitigates Tau Pathology: Inhibiting Tau Uptake and Promoting MTBR‐Tau Clearance through Microglial Pathways in vitro

**DOI:** 10.1002/alz70859_102696

**Published:** 2025-12-25

**Authors:** Miki Murota, Roberta Tufi, Naoki Tokuhara, Hiroshi Ochiai, Tatsuo Horizoe, Yoichi Imaizumi, Malcolm Roberts

**Affiliations:** ^1^ Eisai Co, Tsukuba, Ibaraki Japan; ^2^ Eisai Ltd, Hatfield, Hertfordshire United Kingdom

## Abstract

**Background:**

Tau deposition in the brain is a pathological hallmark of many neurodegenerative disorders that include Alzheimer’s disease (AD). As disease progresses, Braak staging of post‐mortem tissue suggest that pathological tau spreads through the brain via synaptically connected pathways. Tau proteins (full length or fragments) containing the microtubule binding region (MTBR) are essential to form extracellular ‘seeds’ that initiate and propagate pathology. E2814 is a humanised IgG1 antibody that binds to the tau‐MTBR and is currently in clinical trials to prevent the spread of tau pathology in humans. To gain further insight into how mechanistically E2814 (or its murine analogue 7G6) may exert a therapeutic effect, several in vitro neuronal and non‐neuronal (microglia) assays were employed to measure cellular tau uptake.

**Method:**

Tau knock‐in mouse primary cortical neurons and human iPSC‐derived glutamatergic neurons were cultured in vitro. Primary microglia were isolated from mouse brain and human iPSC‐derived microglia were differentiated and maintained by Axol neuroscience (formerly Censo). Recombinant tau proteins were labelled with fluorescent dyes and incubated with various antibodies. Immunocomplexes were added to cells and intracellular tau was monitored using live cell imaging techniques.

**Result:**

In both rodent and human neurons, E2814 and 7G6 reduced levels of neuronal tau uptake over time. In microglia cultures however, an increase in microglial tau uptake was observed which was dependent on effector function of the antibody.

**Conclusion:**

Our results suggest that E2814 may slow down pathological spread of tau by directly preventing tau uptake into neurons and accelerating the removal of MTBR‐tau via microglial clearance pathway.

